# Indispensable Role of Proteases in Plant Innate Immunity

**DOI:** 10.3390/ijms19020629

**Published:** 2018-02-23

**Authors:** Anastasia V. Balakireva, Andrey A. Zamyatnin

**Affiliations:** 1 Institute of Molecular Medicine, Sechenov First Moscow State Medical University, 8, Trubetskaya Str., Moscow 119991, Russia; balakireva.anastacia@gmail.com; 2 Belozersky Institute of Physico-Chemical Biology, Lomonosov Moscow State University, Moscow 119992, Russia

**Keywords:** plant proteases, plant immunity, MTI, ETI, SAR, ISR, RNA silencing

## Abstract

Plant defense is achieved mainly through the induction of microbe-associated molecular patterns (MAMP)-triggered immunity (MTI), effector-triggered immunity (ETI), systemic acquired resistance (SAR), induced systemic resistance (ISR), and RNA silencing. Plant immunity is a highly complex phenomenon with its own unique features that have emerged as a result of the arms race between plants and pathogens. However, the regulation of these processes is the same for all living organisms, including plants, and is controlled by proteases. Different families of plant proteases are involved in every type of immunity: some of the proteases that are covered in this review participate in MTI, affecting stomatal closure and callose deposition. A large number of proteases act in the apoplast, contributing to ETI by managing extracellular defense. A vast majority of the endogenous proteases discussed in this review are associated with the programmed cell death (PCD) of the infected cells and exhibit caspase-like activities. The synthesis of signal molecules, such as salicylic acid, jasmonic acid, and ethylene, and their signaling pathways, are regulated by endogenous proteases that affect the induction of pathogenesis-related genes and SAR or ISR establishment. A number of proteases are associated with herbivore defense. In this review, we summarize the data concerning identified plant endogenous proteases, their effect on plant-pathogen interactions, their subcellular localization, and their functional properties, if available, and we attribute a role in the different types and stages of innate immunity for each of the proteases covered.

## 1. Introduction

Plants are continuously attacked by phytopathogens and have developed various strategies to counter them [1]. Today, the most studied type of immunity in living organisms is the immune system of animals. This is highly complex system and has its own distinct features, such as a highly exquisite adaptive immune structure with an infinite number of antigen-binding receptors that circulate throughout the whole organism and are generated in lymphocytes when a pathogen is encountered [2]. Through the generation of long-lived memory cells, this immune system remembers all antigens that have ever been encountered and multiplies the number of lymphocytes that express such specific antigen-binding receptors, thus allowing the secondary immune response to be faster and more effective [3]. The plant immune system is also very sophisticated, even though compared to the animal system it contains crucial elements, such as high specificity, low self-reactivity, and long-lasting memory, which use unique strategies that seem to have originated independently from animal strategies [4]. Plants have no circulatory system but they do possess cell walls, which are particularly significant in determining the unique features of plant defense.

Plant defense is mainly achieved through the induction of microbe-associated molecular patterns (MAMP)-triggered immunity (MTI), effector-triggered immunity (ETI), systemic acquired resistance (SAR), induced systemic resistance (ISR), and RNA silencing [5]. The development of plant immunity strategies was inextricably intertwined with pathogen strategies and directly depended on them [1]: coevolution and the arms race between plants and pathogens resulted in sophisticated immune responses that need to be tightly regulated. Endogenous plant proteases play an important role in the orchestration of immune processes [6]. Plant genomes encode vast numbers of proteases: the degradome of *Arabidopsis thaliana* L. contains more than 800 proteases from 60 families and the degradome of rice (*Oryza sativa* L.) contains more than 600 proteases [7]. The main function of proteases is proteolysis. Proteases degrade misfolded, damaged and harmful proteins and supply cells with amino acids. Proteases carry out both limited and digestive proteolysis, implying a gain or a switch of function and a loss of function of the proteins, respectively [8]. This makes proteases the major players in the maintenance of cell homeostasis. In addition, proteases also play a regulatory role in a variety of processes that are essential for growth, development, reproduction, immune response, embryogenesis, photosynthesis, programmed cell death (PCD), etc. [7]. Proteases are commonly synthesized as zymogens that determine the folding and function of mature protease. According to the MEROPS database [9], plant proteases are divided into seven classes: serine, cysteine, aspartic, asparagine, threonine, glutamate, and metalloproteases. Serine proteases are the most abundant proteases in plants: they comprise 14 families and nine clans [9]. S8 family contains subtilases that are to date the best-described serine proteases. Serine proteases participate in numerous crucial for vital activity processes such as immunity, symbiosis, PCD, cell differentiation, etc. [10] Cysteine proteases are divided into 15 families of five clans [9]: CA and CE clans contain papain-like folded proteases, while CD clan contains caspase-like folded proteases [7]. Cysteine proteases play an important role in PCD and in responses to biotic and abiotic stresses [11], flowering [12], embryogenesis [7], etc. Metalloproteases are involved in nodulation, plastid degradation, tolerance to stress temperatures, regulation of meristem growth, and meiosis [7,13]. The function of aspartic proteases is not yet well elucidated; however, it is assumed that they are implicated in aging processes [14], plant reproduction [15], and response to stress [16]. *A. thaliana* threonine protease from T1 family subunit beta type (PBA1) is a β-subunit of 26S proteasome and participates in ubiquitin-dependent protein degradation [17]. Glutamate and asparagine proteases have not been studied enough to determine their role in plants. It is noteworthy, that plant proteases are widely used in biotechnology and biomedicine due to their unique features such as wide range of working temperatures and pH values. Among biomedical applications of plant proteases there are antitumor therapy, blood coagulation, wound and burn healing, oral healthcare and treatment of digestive disorders [18].

In terms of immunity, proteases from different families have been shown to participate in almost every stage of immunity establishment, beginning with the pathogen encounter in the apoplast and finishing with their involvement in SAR and transgenerational immune memory [6]. Thus, the aim of the present review is to address the role of plant proteases and their contribution at different stages of distinct types of immunity.

## 2. The Role of Endogenous Plant Proteases in Different Types of Immunity

### 2.1. Plant Immunity Overview

**Figure 1:**
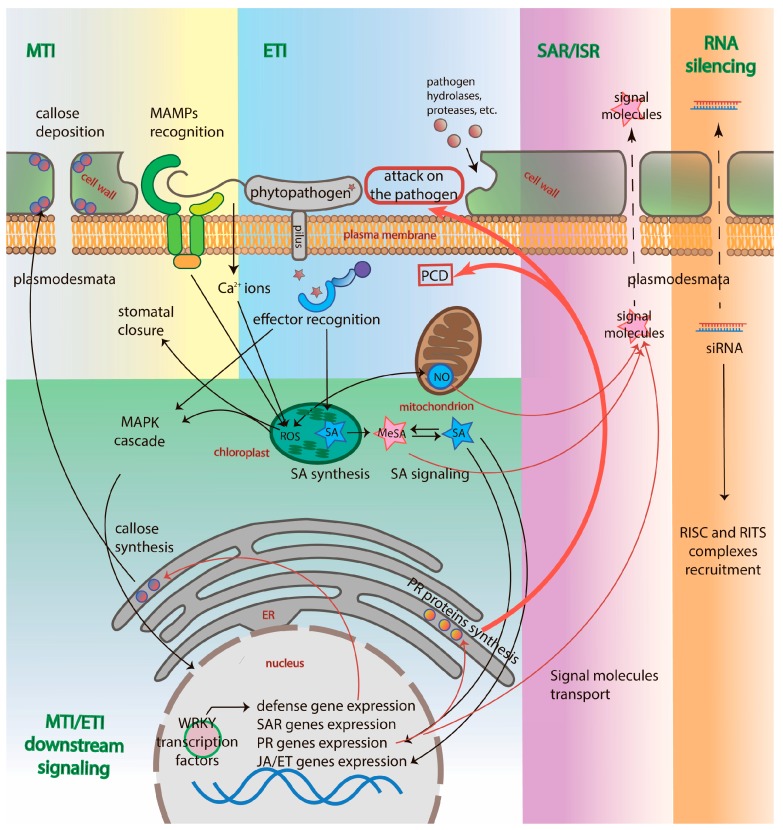
Plant defense mechanisms. MTI is triggered by MAMPs, leading to the elevation of cytosolic calcium ions, ROS and RNI generation, callose deposition at plasmodesmata and stomatal closure [39]. Effectors trigger ETI through binding to R proteins (NB-LRRs) that induce the signaling of SA and the subsequent induction of PR, JA/ET-dependent ISR-related genes and SAR-related genes. PR proteins, such as chitinases, β-1,3-glucanases, proteases, etc., either directly attack the pathogen or induce the PCD of the infected cell. SA is converted into MeSA that is transported into distal parts of the plant, as well as other signal molecules, establishing SAR or ISR. siRNA are also transported into distal parts of the plant through plasmodesmata. Names of immune processes are colored green. The names of cellular compartments are colored red. Black arrows indicate the directions of the activated plant immunity signaling pathways; red arrows indicate the results of genes expression after immunity activation; red bold arrows point to the cell fate in response to the pathogen; dotted arrows indicate the transport of signaling molecules through plasmodesmata.

The first event in plant immunity activation occurs when a cell encounters a phytopathogen. Phytopathogens produce a limited number of microbe- (pathogen-) associated molecular patterns (MAMPs [PAMPs]), such as chitin oligomers, flagellin, lipopolysaccharides and peptidoglycans, that are recognized by pattern-recognition receptors (PRRs) [19]. These receptors contain leucine-rich repeats (LRR) and are receptor-like kinases or proteins (RLKs or RLPs). Such plant PRRs are very similar to the Toll-like receptors (TLRs) found in animal analogs and both plant and animal LRR RLKs are able to recognize different epitopes from the same protein, e.g., flagellin [20,21]. These, in particular, support the convergent evolution hypothesis of the receptors in plants and animals and the independent emergence of pattern-triggered immunity in different domains [22]. PRRs bind to MAMPs through the LRR domain and induce the basal defense or MTI that leads to the activation of a mitogen-activated protein kinase (MAPK) cascade, resulting in the expression of defense genes (Figure 1). These also provide biochemical changes, such as reactive oxygen species (ROS) and reactive nitrogen intermediates (RNI) generation, an increase in Ca^2+^ concentration in the extracellular compartments, etc., and structural improvements, such as stomata closure and callose deposition at the plasmodesmata [23] that hardens the cell wall and hinders pathogen intervention at the site of the pathogen attack (Figure 1) [24].

However, MTI can be overcome by pathogens. First, only a very small number of virulent molecules trigger it and, second, pathogens have developed a way to avoid the MTI system through the emission of effector molecules that directly enter the cell. These effector molecules are “avirulent” signals for PRRs and affect the immunity-associated proteins inside the cell. Thus, well-known effectors, such as AvrRpm1, AvrB, AvrRpt2 in *Pseudomonas syringae*, target the RIN4 protein of *A. thaliana* [5]. Perturbations in the RIN4 protein state are monitored by host-resistance proteins (R proteins) that then recognize the effectors that induce such perturbations [25]. This phenomenon is referred to as the Guard Model, where R proteins are the guardees of targeted by pathogen effector proteins, or the Decoy Model, in which the guarded target acts as a co-receptor to the guardee when it is present and is inactive when the guardee is absent [26]. Large amounts of R proteins exist in plants: pathogen effectors are polymorphic in different organisms and R proteins are specific to them [27]. R proteins contain variable coil-coiled N-terminus, nucleotide-binding site (NBS), and the C-terminal LRR domain, which makes R proteins similar to animal nucleotide-oligomerization domain (NOD)-like receptor (NLR) proteins [28]. The ligand binding activity of the R proteins is followed by conformational changes that are mediated by chaperones HSP90 [29]. Active R protein induces ETI—another type of plant immunity that emerged as a result of the arms race between phytopathogens and plants.

It is worth mentioning that the first contact with a pathogen occurs in the apoplast (extracellular space) of the cell surface that is filled with systemically or locally expressed proteases [30]. Although almost all of the apoplastic proteases belong to the papain-like cysteine protease (PLCP) family, C1A, different proteases represent different pathways that could be attributed to either MTI or ETI and the apoplast battleground is crucial for initial pathogen recognition and for further signal transduction for the establishment of different types or stages of immunity.

The ETI transmits the signal further: it activates the biosynthesis of jasmonic acid/ethylene (JA/ET) and salicylic acid (SA) in chloroplasts that trigger the gene expression of the pathogenesis-related (PR) proteins, and SAR-related proteins (Figure 1). ETI often leads to a hypersensitive response (HR)—a local resistance in the infected site. The HR results in the synthesis of PR antimicrobial molecules, such as chitinase and β-1,3-glucanase, and, if such molecules do not succeed, in the programmed cell death (PCD) of the infected cells [31]. It is worth mentioning that phytopathogens use two different strategies when infecting a plant cell: feeding on the living plant cell or destroying the plant cell to feed on its contents. The PCD of the cell infected by the biotrophic pathogens that feed on living cells, such as viruses, bacteria, fungi, nematodes, and oomycetes, is an effective defense strategy. It is well known that caspases are absent in plants, thus, PCD regulation is attributed to proteolytic enzymes from different families, such as the β-subunit of 26S proteasome PBA1 [17], metacaspases [32], PLCPs [33], vacuolar processing enzymes (VPEs) [34], subtilases [35], etc. [11]. Corresponding proteolytic enzymes and, in particular, their involvement in ETI and PCD will be discussed further.

Induced resistance of plants is presented in two forms: SAR and ISR. SAR is primarily associated with SA-dependent signaling, whereas ISR with JA/ET signaling independent of SA [36]. Both types of induced resistance make uninfected plant parts more resistant towards a broad spectrum of plant pathogens. Despite the lack of a plant circulatory system, mobile immune signals may be transmitted from the ETI-associated local resistance infected site to distal healthy areas, inducing SAR in order to protect the whole plant organism from the invader. In plants infected with the Tobacco mosaic virus (TMV), *Nicotiana benthamiana* L., SAR lasted for 20 days against TMV and other pathogens [37]. Immune signals travel through the apoplast and the phloem and include methylsalicylic acid (MeSA), lipid-derived compounds azelaic acid and JA, and glycerol-3-phosphate [22]. The lipid-transfer proteins, Defective in Induced Resistance 1 (DIR1) and Azelaic Acid Induced 1 (AZI1), transport mobile immune molecules [38]. As a result, MeSA is converted back into SA and the accumulation of SA in the phloem occurs, thus establishing SAR. Subsequently, SA accumulation leads to the induction of immune-related genes (PR proteins) in distal tissues: the synthesis of β-1,3-glucanase, chitinase, defensins, proteases, etc., and transgenerational immune memory (Figure 1) [22].

SAR induces total cell reprogramming: expression of 14 classes of PR proteins, including proteases, mainly regulated by Nonexpresser of PR genes 1 (NPR1) [40] and conferring long-lasting immunity through cell priming—a sensitized state that enables a faster and more effective response to a secondary pathogen attack. Cell priming is thought to be connected to MAPK accumulation, MPK3 and MPK6 [41]. In addition, *R* genes are known to form clusters, thus it was proposed that the transgenerational immune memory is achieved through the duplication events of these genes and the hypomethylation of the chromatin, where these clusters are located, after infection [42].

ISR as well as JA/ET signaling is associated with defense against herbivores [43] and rhizobacteria [36]. ISR does not imply synthesis of PR proteins and SA and could be induced by PAMPs. In *A. thaliana*, SAR is most effective against biotrophic pathogens, downy and powdery mildews, as well as viruses that are sensitive to SA-dependent defenses; SAR inhibits plant growth whereas ISR is more active against nectrotrophic pathogens and promotes plant growth [36]*.* RNA silencing is a defense pathway of plant immunity that deals with viruses and fungi [44]. RNA silencing implies the use of small regulating RNAs (siRNAs) to specifically inactivate the nucleic acids of the pathogen [45]. This is divided into two directions: transcriptional gene silencing (TGS) and post-transcriptional gene silencing (PTGS) [46]. PTGS implies the elimination of viral mRNA by RNA-induced silencing complex (RISC) through the formation of the siRNA-mRNA complex. siRNAs are generated by Dicer through the cleavage of dsRNA synthesized from the viral genome of RNA-dependent RNA polymerase (RNA viruses) or RNA polymerase II (DNA viruses) [46]. DNA cytosine methylation occurs during TGS: a prime epigenetic event in the defense response to viruses [47]. Heterochromatic ssRNAs are produced by RNA polymerase IV, which is converted into dsRNA, diced by RNA-dependent RNA polymerase 2 (RDR2) and incorporated into the RNA-induced transcriptional silencing complex (RITS complex) that acts as a guiding strand for viral heterochromatin formation and methylation [48]. It is interesting to note that RNA silencing signal factors (ssRNAs and microRNAs) can travel from cell to cell through plasmodesmata and to distal parts of the plant through the phloem, just like MeSA molecules establishing SAR (Figure 1) [49].

### 2.2. Involvement of Endogenous Plant Proteases in Different Types of Plant Defense

#### 2.2.1. Plant Proteases Functionality in Immunity Establishment

Proteases perform different functions in respect of plant defense. First, proteases activate different signaling processes by carrying out PRRs and NB-LRRs controlled proteolysis, also known as ectodomain shedding [50]. Although the precise mechanisms of protease action and their substrates are yet not well elucidated, the only known example of ectodomain shedding is the cleavage of the chitin receptor of *A. thaliana*, Chitin Elicitor Receptor Kinase 1 (CERK1), by an obscure protease [51].

Second, proteases are able to release signaling peptides that are perceived as damage-associated molecular patterns (DAMPs) by PRRs and induce immunity. For example, the aspartic protease, Constitutive Disease Resistance 1 (CDR1), generates PAMP or hormone systemin-like peptides that activate basal immunity [52]. Moreover, the recombinant prodomain sequences of C1A proteases from barley, can, alone, control phytophagous arthropods (coleopteran and acari) and reduce leaf damage [53].

**Figure 2:**
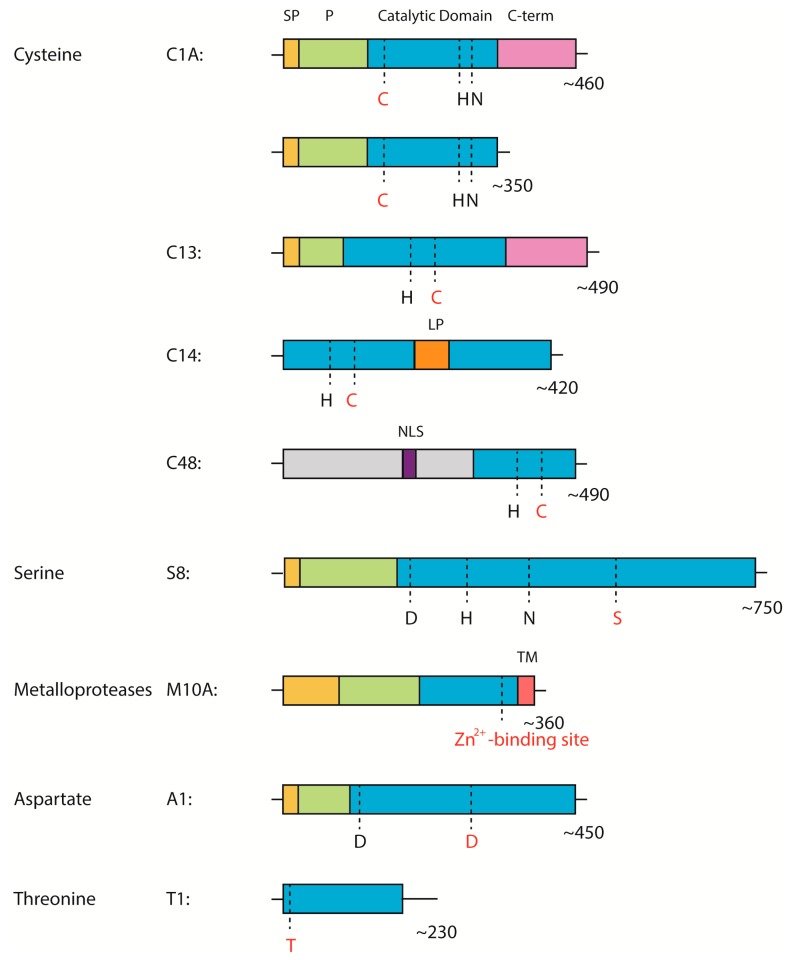
The domain architecture of proteases covered in the review from different protease families. Red letters represent catalytic amino acid residues. SP—signal peptide, P—prodomain, C-term—C-terminal domain or granulin domain (for family C1A proteases), LP—linker peptide, NLS—nuclear localization signal, TM—transmembrane region, regions in grey—low complexity regions.

Third, proteases orchestrate and regulate a large amount of the signaling pathways of MTI, ETI, SAR, ISR, and the RNA silencing of plant defenses. We focus on this aspect of protease action in the review. Despite the fact that a number of proteases have already been identified as being in some way involved in plant immunity, the exact mechanisms of the majority of proteases action, their substrates, and the signaling pathways they involve remain a mystery. The authors collected all the available data on endogenous plant proteases from the different families that implement different catalytic mechanisms, their functions during plant defense establishment, and their domain architectures (Figure 2) and attempted to attribute the proteases to the different signaling pathways of immunity (Table 1 and Table 2). However, it is worth mentioning that in the case of MTI and ETI, the signals received from both pathways through PRRs or NB-LRRs line up in the same MAPK cascade and synthesis of mobile signaling molecules (Figure 1). Thus, one endogenous protease may participate in different types of defense or in shared parts of the pathways. Nevertheless, plant immunity is still a highly complex and obscure field of research.

#### 2.2.2. Proteases Involved in MTI

As described above, endogenous proteases can produce MAMP- (PAMP-), DAMP-like peptides that are perceived by PRRs. The *Glycine max* L. cryptic signal, 12-aa peptide GmSubPep, was shown to be part of a unique subtilase; the peptide acts as PAMP and causes a pH increase, inducing immunity response when supplied to soybean cultures by induction of the expression of known defense-related genes, such as Cyp93A1, Chib-1b, PDR12, and *achs* [54]. Another fascinating example of the peptide cleavage of a protease is a major hub of the flg22 (flagellin epitope, MAMP)-regulated transcriptional network propeptide of Rapid ALkalinization Factor 23 (proRALF23), confirmed to be a substrate of subtilase SBT6.1 (S1P) in *A. thaliana* [55]. The proRALF23 cleavage of S1P results in the dose-dependent inhibition of elf18 (EF-Tu epitope, MAMP)-induced ROS production and the scaffolding function of EF-Tu receptor (EFR) (the receptor of elf18 that increases the susceptibility to *Pseudomonas syringae* pv. *tomato* DC30000 (*Pst*DC3000)), which makes S1P a negative regulator of plant immunity. The aspartic protease, CDR1, is also able to generate PAMP- or systemin-like peptides and induces the basal defense response and SAR [52].

PRRs might also be targets of plant proteases and not only in terms of ectodomain shedding. Tomato S8 subtilase P69C, which derives from tomato, is able to process the extracellular LRP protein that is possibly involved in immunity [56]. *A. thaliana* cysteine proteases from the family C19 ubiquitin carboxyl-terminal hydrolase 12 and 13 (AtUBP12 and AtUBP13), appear to be negative regulators of plant immunity: they are able to suppress Cf-9-mediated HR in *N. benthamiana* following *Pst*DC3000 infection through the possible deubiquitinylation of PRRs [57].

A conventional sign of MTI is callose accumulation in the plasmodesmata that blocks symplast transport and isolate the infected cells [23]. This occurs following recognition of the flg22 by the Flagellin-Sensing 2 (FLS2)-Brassinosteroid Insensitive 1-associated receptor Kinase 1 (BAK1)-*Botrytis*-Induced Kinase 1 (BIK1) complex and the ROS generation that promotes regulated callose synthesis, which occurs rather not through the one conserved downstream cascade, but its own unique multiple mechanisms [58]. The overexpression of the aspartate protease 13 from *Vitis quinquangularis* (VqAP13) in *A. thaliana* plants increased resistance to *Pst*DC3000 and accumulated more callose in the infected site than in wild-type plants, suggesting the involvement of VqAP13 in MTI signaling. The expression of VqAP13 was upregulated by SA and ET treatment and downregulated by MeJA and *Botritis cinerea* infection, suggesting the participation of the protease in defense against biotrophic pathogen [59].

Another sign of MTI is stomata closure that occurs in response to the elevation of cytosolic Ca^2+^ concentrations and the subsequent H_2_O_2_ and NO accumulation in the guard cells [60]. VPE cysteine proteases are essential for elicitor-induced stomatal closure in *N. benthamiana*: VPEs are localized in the vacuole and control the fusion of the plasma membrane and the vacuole membrane during virus-induced PCD [61]. VPE mediates elicitor-induced stomatal closure by regulating NO accumulation in the guard cells [62]. One study has recently reported that the plant pathogen, *Pst*DC3000, uses the virulence factor coronatine (COR) to actively open stomata, as well as oxalate, which is produced by many fungi species [63]. This makes VPE-dependent stomatal closure an important defense mechanism.

#### 2.2.3. Proteases Involved in ETI, PCD, and RNA Silencing

Effectors have emerged as a result of an arms race between plant MTI and pathogen strategies developed to avoid it. Pathogens emit avirulent molecules—effectors—that are primarily dedicated to the activation of host transcription, acting as transcription factors, in order to affect chromatin and histone packaging and to regulate nutrient release for pathogen survival [5]. In addition, it has been concluded that a single dominant host-resistance gene (*R*) incites a phenotype of disease resistance in response to a pathogen expressing a single dominant avirulence gene (*Avr*) [64]. This model was known as the gene-for-gene hypothesis, implying that each dominant pathogen avirulence (*Avr*) gene product is either directly or indirectly recognized by the product of a corresponding dominant host *Cf* resistance gene. For example, in the *Cladopsorium fulvum*-tomato interaction, five *Avr* genes (*Avr2*, *Avr4*, *Avr4E*, *Avr5*, and *Avr9*) have been characterized from *C. fulvum* and their encoded proteins trigger an HR in host plants carrying the corresponding *Cf-2*, *Cf-4*, *Cf-4E*, *Cf-5*, and *Cf-9* genes, respectively [65].

Plant proteases have been shown to participate in Avr/Cf-induced HR (Table 1). *Phytophthora*-inhibited protease 1, from *N. benthamiana*, NbPip1, is associated with an Avr4/Cf-4-induced HR; in NbPip1 mutant HR was delayed for one to two days [66], whereas Rcr3 from tomato acts as co-receptor to Cf-2 for Avr2 effector recognition [67]. *Cf*-encoded R proteins are NB-LRRs that mostly act in the nucleus for the direct rapid regulation of gene expression through the activation of immune responses [68]. The indirect recognition of the effectors is managed by the RIN4 protein that is targeted by many bacterial effectors (AvrRpt2, AvrRpm1, AvrB, and HopF2) and is monitored by NB-LRRs ribose-phosphate pyrophosphokinase PRS2 and disease resistance protein RPM1 that activate ETI in the case of changes to the RIN4 state [69].

The ETI response subsequently activates the MAPK cascade that induces transcription from the PR genes. This induces the biosynthesis of SA, JA, and ET and antimicrobial enzymes, as described above. Degradation of NPR1 is a molecular switch that depends on its paralogues NPR3 and NPR4, receptors of SA, that bind it with different affinities and regulate degradation of NPR1 in SA-regulated manner [100]. NPR3 and NPR4 are adaptors of the Cullin 3 ubiquitin E3 ligase, and *npr3 npr4* double mutant plants accumulate a high amount of NPR1 protein and are not able to launch ETI, PCD, or establish SAR. Plant PCD is characterized by chromatin condensation, shrinkage of cytoplasm, swelling of mitochondria, vacuolization, and chloroplast disruption [101]. The chloroplast plays a central role in the defense responses and HR of plants. It is a source of defense signaling molecules, such as ROS, RNI, SA, and JA [102]. In addition, pathogen effectors that possess chloroplast localization signals suppress immunity [103].

**Figure 3:**
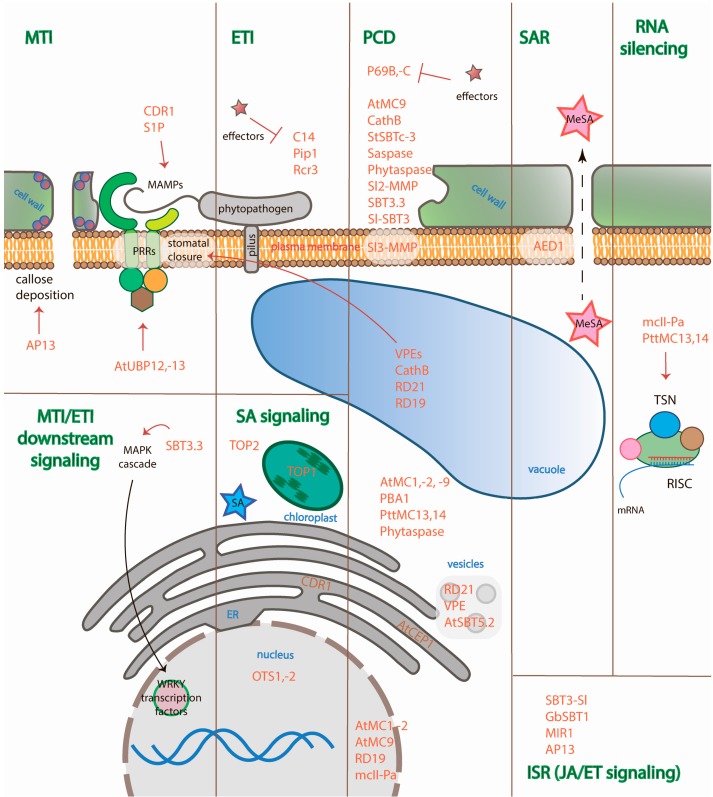
Involvement of plant proteases in different immunity pathways and their subcellular localization. Proteases covered in the review are colored orange. Immune processes are colored green. The names of cellular compartments are colored blue. Red arrows point to the object of the protease action (hydrolysis of the substrate or influence on the process); T-like arrows imply inhibition of proteases by effectors; black arrow indicates the direction of the MAPK cascade action; dotted arrow indicates the transport of signaling molecules through plasmodesmata.

Metacaspases are associated with PCD and RNA silencing in plants. When infected with a necrotrophic pathogen, infection leads to the necrosis of the cell, whereas when infected with a biotrophic pathogen, the plant organism launches PCD to eliminate the infected cells. However, at the same time, such pathogens suppress PCD to feed on living cells. The main executioners of PCD in animals are caspases—cysteine aspartate-specific proteases that are absent in plants—and PCD in plants is associated with caspase-like activities but not with caspases (Table 1). Metacaspases are very distant homologs of caspases and their biochemical properties also differ: their substrate specificity is arginyl-/lysil-specific, whereas caspases are aspartate-specific proteases. Metacaspases are divided into types I (AtMC1-AtMC3) and II (AtMC4-AtMC9). Type I metacaspases contain an additional N-terminal proline-rich prodomain with a zinc finger motif [11]. *A. thaliana* metacaspases type I, AtMC1 and AtMC2, have been shown to regulate autolytic PCD (involving rapid cytoplasm clearance): they suppress the hypersensitive cell death response upon infection with the avirulent pathogen, *Pst*DC3000. AtMC1 and AtMC2 antagonistically control Lesion Simulating Disease 1 (LSD1) runaway cell death [32]. The *A. thaliana* type II metacaspase, AtMC9, functions extracellularly during the autolysis of xylem elements after their cell death: it provides post-mortem clearance of the cell contents after vacuolar rupture [70]. In extracellular space, AtSerpin1 inhibits AtMC9 [104]. AtMC9 has been shown to cleave 11 amino acids from GRI protein involved in PCD initiation [72]. Another substrate of AtMC9 is phosphoenolpyruvate carboxykinase 1 (PEPCK1)—one of the components of gluconeogenesis in plants. PEPCK1 cleavage by AtMC9 leads to the enhancement of enzyme activity, suggesting the role of AtMC9 in limited proteolysis and its effector properties [71]. The homologs of AtMC9 from hybrid aspen (*P. tremula* x *tremuloides*), PttMC13 and PttMC14, were shown to be involved in a variety of xylem processes: in the metacaspase-Tudor Staphylococcal Nuclease (TSN) pathway, in the regulatory proteolysis of Responsive To Dehydration 21 (RD21) during xylem maturation, and in the cell death of xylem elements (PASPA3 aspartate protease processing) [90]. They are also expressed during the non-autolytic type of PCD, i.e., vacuolar collapse [90]. It is worth mentioning that although caspases and metacaspases possess different substrate specificities and biochemical properties, they are functionally similar: both proteases can hydrolyze a single conserved substrate which is very important for the vital activities of different species. It has been shown that the protein TSN, which is found in plants and animals (including humans) and is essential for transcription, RNA splicing, RNA editing, etc., is a substrate for both caspase-3 and Norway spruce metacaspase type II (mcII-Pa) [81,105]. TSN manages activation of transcription, activation of mRNA splicing, regulation of RNA silencing as a member of RISC complex [105]. TSN was shown to be an anti-apoptotic agent: uncleavable TSN stimulates cell proliferation and protects cells from death. Degradation of TSN by mcII-Pa results in impairment of its ability to activate mRNA splicing, inhibition of its ribonuclease activity, and in PCD of the cell. mcII-Pa was shown to execute PCD of embryo suspensor cells during plant embryogenesis [81]. These data suggest that TSN cleavage by mcII-Pa represents a regulatory mechanism that limits RNA silencing and promotes PCD of the cells infected by virus (Figure 3).

Vacuole collapse is a type of PCD that is characterized by the absence of rapid cytoplasm clearance and is held by cysteine proteases—VPE enzymes [34]. VPEs exhibit caspase-1-like activity and substrate specificity toward an asparagine residue; they localize in the vacuolar membrane and mediate virus-induced hypersensitive cell death by regulating the collapse of the vacuole membrane and the release of vacuolar hydrolytic enzymes for attacking the virus in response to infection. VPEs possess YVADase activity to mediate TMV-induced PCD [62]. Another protease—the threonine-dependent PBA1, β-subunit of proteasome—orchestrates a further vacuole-associated defense mechanism: proteasome-regulating membrane fusion of the vacuolar and plasma membranes that provides plants with a mechanism for attacking intercellular bacterial pathogens. Following bacterial infection, proteasome-regulating vacuolar-plasma membrane fusion occurs in the intact cell wall, resulting in the discharge of the vacuolar contents in the apoplast—the extracellular antibacterial fluid that contains PBA1 manifests caspase-3-like DEVDase activity and mediates the consequent PCD [17].

Subtilases (serine proteases, family S8) also exhibit caspase-like activity and contribute to plant PCD. Tomato S8 subtilases, P69B and P69C, were the first identified plant apoplastic proteases. These subtilases are PR proteins that provide basal levels of surveillance; their promoters are induced by SA and they are synthesized as preproenzymes, both locally (in the infected site) and systemically (in distal, non-infected sites), in response to *P. infestans* and *P. syringae* [106]. The P69B protease is targeted and inhibited by the Kazal-like inhibitors EPI1 and EPI10 from *P. infestans* [83]. P69B was recently shown, in vitro, to be a substrate of the tomato matrix metalloproteases Sl2- and Sl3-MMPs. The overexpression of P69B leads to the PCD of the epidermis cells of tomato hypocotyls, suggesting that P69B is a positive regulator and Sl2- and Sl3-MMPs are negative regulators of the PCD of the epidermis [84]. Sl2- and Sl3-MMPs are also secreted into the apoplast, they belong to the M10A family of metalloproteases and are synthesized in the form of preproenzymes.

Saspases from oats possess IETDase and LEHDase activities and cleave RuBisCO molecules during chloroplast rapture in victorin-induced PCD (toxin from *C. victoriae*) [91], whereas phytaspases exhibit VEIDase, IETDase, LEHDase, and VDVADase activities and are activated during TMV-induced PCD [92]. Saspases are constitutively expressed in an inactive form; processed and relocalized into the apoplast following PCD induction. Although the exact role of saspases remains obscure, phytaspases cleave off the nuclear localization signal (NLS) from NLS-containing the *A. tumefasciens* protein, T-DNA border endonuclease VirD2, which is essential for the nuclear uptake of foreign DNA within the plant cell during bacterial infection and plant transformation [107]. Phytaspases are constitutively synthesized and located in an active form in the apoplast in normal conditions and are relocalized into cytoplasm upon PCD induction [92,108,109]. Another subtilase, from *S. tuberosum*, StSBTc-3, was shown to possess caspase-3-like—DEVDase—activity. It is located in the apoplast and accumulated in detached leaves following *P. infestans* infection. This protease is constitutively expressed and, in vitro, may induce PCD and cytoplasm shrinkage [110]. The subtilase AtSBT5.2, from *A. thaliana*, was shown to be a negative regulator of MYB30 activity—a transcription factor that promotes the cell death-associated response activation of genes that participate in lipid biosynthesis [80]. The variant protein, AtSBT5.2(b), retains MYB30 in vesicles that are not allowed to enter a nucleus during infection.

PLCPs have also been shown to regulate plant PCD. The vast majority of studied apoplastic proteases are represented by papain-like cysteine proteases (PLCP) that comprise the C1A subfamily of cysteine proteases. The C1A family was subclassified by Richau [111] into nine classes according to their homology and domain architecture. These classes are combined into four groups in accordance with their similarity to human cathepsins: CathL-like, CathF-like, CathH-like, and CathB-like. All of the apoplastic PLCPs described below are CathL-like proteases. The accumulated data on PLCPs specificities suggest that they have a rather low specificity. However, nonpolar (including Pro) or aromatic amino acid residue was found to be preferential at the P2 position of the substrate [112,113,114,115]. PLCP are key players in a variety of processes, including growth, development, responses to stresses and defense. PLCP are upregulated in the *atg* mutants (autophagy mutants) of *A. thaliana*, suggesting either their backup role in nutrient recycling and remobilization or their cell-death promoting role [116].

In plant immunity, in contrast to subtilases, pathogen effectors inhibit apoplastic PLCPs that are strongly associated with the ETI response. For example, tomato and potato RD21-like C14 protease are inhibited by the cystatin-like effectors EPICs [117] and the AVRblb2 effector from *P. infestans* [88]. C14 is synthesized as preproenzyme and contains a unique granulin domain with an unknown function (Figure 2). It is processed through cleavage of the prodomain (iC14 form) and the subsequent granulin domain cleavage (mC14 form). The silencing of C14 leads to susceptibility to *P. infestans* in *N. benthamiana* C14 is a highly conserved protease; it is targeted by pathogen effectors and its homologs are present in different species of the plant kingdom, e.g., in tomato Cysteine Protease 1 (CYP1)—the orthologue of C14 that does not contain granulin domain and is targeted by the V2 protein of the tomato yellow leaf curl virus (TYLCV) [87]. Cysteine proteases CP1A, CP1B, CP2, and XCP2 from maize are also orthologues of C14, they do not contain the granulin domain and are targeted by the Pit2 effector of *Ustilago maydis* [118]. A lack of these enzymes results in increased susceptibility to pathogens.

Fascinating examples of the coevolution of pathogen effectors and plant defenses, driven by the arms race, are tomato paralogous apoplastic Senescence-Associated Gene 12 (SAG12)-like PLCPs RCR3 and *Phytophthora*-inhibited protease 1 (PIP1), which are targeted by the different effectors of different pathogens, suggesting the importance of these proteases for plant defense [85]. Both proteases are expressed constitutively in leaves but their expression increases after inoculation with *C. fulvum* (both virulent and avirulent strains), *P. infestans*, and *P. syringae*. Both proteases are targeted by the effector Avr2 and the cystatin-like effectors, EPICs [86]. RCR3 is also targeted by the nematode effector, Gr-VAP1 [67]. PIP1 expression is 10× higher than RCR3, and this is a major broad-range immune protease against various apoplastic pathogens: the absence of PIP1 leads to hyper-susceptibility to fungal, bacterial and oomycete plant pathogens, whereas RCR3 acts as a co-receptor to the immune receptor Cf-2 for the recognition of Avr2. The absence of Rcr3 causes increased susceptibility only to the *P. infestans*. The functional divergence of paralogous genes provides more effective defense against different pathogens. In addition, a homolog of the tomato PIP1, NbPip1 from *N. benthamiana*, contributes to the Avr4/Cf-4 induced hypersensitive response [66].

Non-apoplastic PLCPs are associated with PCD. Cathepsins in animals are lysosomal proteases that are associated with cell death and survival [119,120,121,122]. In *A. thaliana*, AtCathB is likely to be a positive regulator of cell death and basal resistance, mediated through interactions with LSD1. A key marker gene of senescence, SAG12, is downregulated in *atcathb* triple mutants. Thus, cathepsin B is required for HR and for resistance to non-host bacterial pathogens [33].

*A. thaliana* RD21 is known to be a pro-death cysteine protease, which is accumulated in ER bodies in an inactive form. RD21 is a homolog of C14. The proteolytic domain, RD21, also contains a signal peptide, prodomain and granulin domain (Figure 2), which are cleaved during processing: prodomain cleavage results in the intermediate form, iRD21, and granulin domain cleavage, in the mature form, mRD21 [123]. RD21 is physically and reversibly inhibited by the Kunitz-type inhibitor, *A. thaliana* water-soluble chlorophyll protein (AtWSCP), which regulates PCD during flower development [75]. In detached leaves, RD21 is also targeted by the inhibitor, AtSerpin1: AtSerpin1 is localized in cytoplasm, while RD21 is localized in ER bodies, and elicitors of PCD increase the permeability of vesicles membranes, which leads to the colocalization of RD21 and AtSerpin1 in cytoplasm and the irreversible inhibition of the protease. This suggests the pro-survival role of AtSerpin1 [76,124]. In addition, as already mentioned, RD21 is also processed by the type II metacaspases, PttMC13 and PttMC14 [90]. The RD21 mutants are unaffected in interactions with *P. syringae* but are more susceptible to the necrotrophic fungal pathogen, *B. cinerea*, demonstrating that RD21 provides immunity to a necrotrophic pathogen.

The orthologue of RD21, RD19, is also involved in plant defense: it is inhibited by the effector of *R. solanacearum* PopP2 that interacts with the *A. thaliana* Resistant to *R. solanacearum* 1-R (RRS1-R) R protein [77]. RD19 is induced during *R. solanacearum* infection and is required for RRS1-R-mediated resistance: RD19 is normally localized in mobile vacuole-associated ER compartments and, on interaction with the effector PopP2, it is relocalized to the plant nucleus. The nuclear complex RD19-PopP2 is recognized by RRS1-R and is required for the ETI response.

A KDEL-containing cysteine endopeptidase 1 from *A. thaliana* (AtCEP1) is also located in ER and functions during developmental cell death and tissue remodeling. However, it has been shown to be associated with late defense reactions: it restricts the growth of the biotrophic fungus *E. cruciferarum* and participates in late epidermal cell death; it is a possible target for pathogen effectors [79]. AtCEP1 is regulated by hormone molecules rather than by MAMPs, and it accumulates around the haustoria, inducing PCD. AtCEP1 is likely to be delivered from the ER to the vacuole through late endosomes with a subsequent fusion of the vacuole and plasma membranes, resulting in the relocalization of AtCEP1 into the apoplast or extrahaustorial space [17]. KDEL-cysteine endopeptidases, such as AtCEP1, are considered to be late-acting proteases that digest cell wall proteins during the final stages of PCD and tissue remodeling following cellular disintegration [125].

#### 2.2.4. Proteases Involved in MTI/ETI Downstream Signaling Pathways, SAR and ISR

Downstream MTI and ETI events partially overlap and include the activation of the MAPK cascade and WRKY transcription factors that contain the WRKY domain that is defined by the conserved amino acid sequence WRKYGQK at its N-terminal end [126]. WRKY transcription factors manage the rapid activation of the PR genes associated with the biosynthesis of the signal molecules, SA, JA, and ET, with lignifications, the production of antimicrobial agents, etc. [127,128]. The subtilase SBT3.3, from *A. thaliana*, activates MAPKs and the Oxidative Signal-Inducible 1 (OXI1) kinase following pathogen attack (Table 2). In knockout SBT3.3 plants, the MAPK cascade is inhibited, as is chromatin remodeling, suggesting its involvement in the regulation of kinases and in epigenetic regulation [35].

Plants produce SA from chorismic acid through two biosynthetic pathways, one catalyzed by the phenylalanine lyase1 to 4 (AtPAL1–4) and the other by isochorismate synthase1 and 2 (AtICS1 and 2) in chloroplasts [129]. The accumulation of SA leads to the establishment of SAR—a long-lasting broad-spectrum disease resistance that implies the activation of defense mechanisms in infected sites and is targeted to protect distal healthy tissues [130]. SAR leads to the total reprogramming of PR genes and cell priming. This is achieved through the production of the mobile signal molecule, MeSA, in the infected site and its transport, through the plasmodesmata and phloem, to distal parts of the plant [131]. Delivered to healthy cells, MeSA is converted into SA and is bound by its NPR3 and NPR4 receptors, resulting in NPR1 degradation and induction of SAR-related genes [100]. SAR-associated proteases are summarized in Table 2.

SA-activated downstream genes are divided into immediate-early genes and late genes, which include the SAR-marker gene, *pr1* [132]. Thimet oligopeptidases (TOPs) 1 and 2 are metalloproteases from *A. thaliana* that interact with SA, resulting in the loss of their proteolytic activity, both in vitro and in plant extracts [98]. The absence of TOPs results in increased susceptibility to *P. syringae* [98]. It has been suggested that TOPs mediate the SA-dependent signaling pathways and act as modulators of chloroplast and cytosolic PCD-related processes that may not be exclusively activated by pathogen infection.

Overly Tolerant to Salt 1 and 2 *(*OTS1 and 2*)* are cysteine proteases, from *A. thaliana* that cleave off Small Ubiquitin-Related Modifier 1 and 2 (SUMO1 and 2) and manage the SUMO1/2-mediated regulation of SA signaling [96]. Double mutant *ots1/2* displayed enhanced resistance to virulent *P. syringae*, higher levels of SA and upregulated expressions of the *ics1* SA synthetic gene as compared with wild-type plants. SA stimulated OTS1/2 degradation and promoted the accumulation of SUMO1/2 conjugates. The accumulation of SUMO1/2 conjugates coincides with SA-promoted OTS degradation and may play a positive role in SA-mediated signaling.

The apoplastic aspartate protease, Apoplastic EDS1-Dependent protein 1 (AED1) from *A. thaliana*, was shown to be induced locally and systemically by SA and its analog, benzothiadiazole [97]. Enhanced Disease Susceptibility 1 (EDS1) protein is essential, both for SAR signal generation in an infected site and its perception in the systemic tissues. In *eds1* mutant plants, the conditional overaccumulation of AED1-hemagglutinin inhibited SA-induced resistance and SAR (EDS1-dependent), but not local resistance (EDS1-independent). The data suggest that AED1 is part of a homeostatic feedback mechanism that regulates systemic immunity. Another aspartate apoplastic protease is CDR1, from *A. thaliana*, whose hyperactivation induces an SA-dependent disease resistance response [52]. This activates SAR and induces the accumulation of SA and the transcripts of the *pr1* and *pr2* genes, which are markers of SAR. The CDR1 protein is accumulated in the apoplast in response to the inoculation of avirulent bacterial pathogens. CDR1 may generate extracellular peptide elicitors that activate the basal defense response.

JA is a main signal molecule of ISR and is produced through the induction of the octadecanoid pathway. JA is a pleiotropic hormone and JA-dependent genes encode an arsenal of plant defense proteins involved in resistance to insects and necrotrophic pathogens [133]. JA and ET defense signaling pathways are transduced and integrated through the ET-responsive transcription factor superfamily, binding to the GCC box of PR proteins [134]. JA-induced (ISR) and SA-induced (SAR) signaling pathways negatively regulate each other: the SA signaling pathway is involved in the resistance to biotrophic pathogens, while the JA and ET signaling pathways principally mediate resistance to necrotrophic pathogens [135]. However, only a few genes that regulate the interplay between JA and SA signaling networks have been identified (Table 2). The overexpression of the aspartate protease 13, AP13, from *V. quinquangularis*, described above, not only enhances resistance to powdery mildew but also contributes to SA, JA, and ET regulation: its transcript levels decreased following *B. cinerea* (necrotrophic pathogen) infection and MeJA treatment but increased following ET and SA treatments. This suggests that VqAP13 action promotes the SA-dependent signal transduction pathway but suppresses the JA signal transduction pathway. The overexpression of VqAP13 suppresses the biosynthesis of JA, as well as the expression of downstream genes, e.g., lipoxygenase 3 and plant defensin 1.2, and the upregulation of the expression of the genes *ics1* and *pr1*, which are components of the SA biosynthesis pathway and the SA signaling pathways, respectively [59].

The tomato matrix metalloprotease, Sl3-MMP, expression is induced by infection with *B. cinerea*, *Pst*DC3000, and by the SA, JA and ET treatment [93]. Sl2- and Sl3-MMP prefer hydrophobic amino acid residues in the P1 position and proline residue in the P3 position of a substrate [84]. The absence of Sl3-MMP results in increased susceptibility to *B. cinerea*, *Pst*DC3000. In addition, treatment with SA, MeJA, and the precursor of ET 1-amino cyclopropane-1-carboxylic acid (ACC) showed a three to four-fold increase as compared to a control, suggesting the activation of the Sl3-MMP gene in late ETI responses.

The subtilase from *G. barbadense*, GbSBT1, is closely related to the AtSBT5.2 described above and is associated with the defense response to infection by *V. dahliae* [95]. The isochorismatase, VdISC1, is secreted by *V. dahliae* to suppress the SA-mediated innate immunity of host cells. Verticillium wilt resistance is, thus, related to the JA signaling pathway. The GbSBT1 is normally localized in the plasma membrane, whereas after treatment with JA and ET, it is relocalized into the cytoplasm, suggesting the protease to be a sensor of the combined signals of ET and JA treatments and its involvement in ISR. Moreover, the overexpression of GbSBT1 in *A. thaliana* results in the activation of MAPK signaling and JA-responsive genes [136].

Proteases that contribute to ISR and to the defense against insects have also been identified (Table 2). An interesting example of a plant protease that participates in the defense against herbivorous insect attack is cysteine protease MIR1, from maize, which is activated in response to caterpillar feeding [99]. It is ET-dependent and accumulates in maize midwhorl, resulting in a reduction in caterpillar growth and affecting the caterpillar peritrophic matrix. Another protease is subtilase SBT3-Sl, from tomato, which is induced after wounding and insect attack [94]. SBT3-Sl silencing results in decreased resistance to the larvae of the specialist herbivore, *M. sexta*, and attenuates the induction of systemic wound defense genes. SBT3-Sl was found to be stable and active in the insect’s digestive system, from where it may act on unidentified proteins of insect or plant origin. In addition, SBT3 is involved in the regulation of pectin methylesterases.

## 3. Conclusions

To date, plant proteases are known to be crucial components of plant immunity. In this review, we have summarized the available data on the proteases of different plant species and participation of the proteases in immune processes. We have also attempted to classify the proteases according to their involvement in different types and at different stages of plant defense. It is worth mentioning that proteases seem to be implemented in every step of immunity establishment: pathogen encounter, generation of DAMPs and MAMPs, effector recognition, regulation of PRRs and NB-LRRs (R proteins) action, signal transduction (including MAPK cascade activation), involvement in the synthesis of signal molecules, the orchestration of PCD, cell priming, the regulation of PR proteins expression, SAR and ISR establishment, and, finally, RNA silencing. It is noteworthy that proteases are extremely powerful tools in any organism and tight regulation of their action is required. Pathogen effectors that target plant proteases are mainly represented by protease inhibitors [137] but the plant itself synthetizes inhibitors to regulate their activity. For example, an irreversible inhibitor, AtSerpin1, and a reversible Kunitz-type protease inhibitor, AtWSCP, tightly regulate the activity of the *A. thaliana* cysteine protease, RD21, in plant development and defense [124]. In addition, the production of maize CC9 cystatin inhibitor, which is induced by an *U. maydis* infection simultaneous with the production of cysteine proteases, results in the inhibition of the proteases in the apoplast; the pathogen manipulates the transcription of the plant inhibitors to facilitate infection [138].

Moreover, plant proteases possess unique features that could be applied in biotechnology and biomedicine, e.g., papain from *Carica papaya* L. and bromelain from *Ananas comosus* L. are already used in very different fields of industry. However, the question concerning the role of plant proteases in the immune system and in vital processes of plant organisms, in general, is still highly relevant and remains a mystery. Nowadays, more and more complete plant proteomes are becoming available. With the use of predictive bioinformatic tools relying on already existing data, it has become a great opportunity to identify novel enzymes, including proteases. Identified proteases could be studied through the investigation of generated mutant plants carrying loss-of-function (or other) mutations in the corresponding genes. In addition, the knowledge concerning already known proteases could be expanded further: nowadays, a little data on the substrate specificity and functional substrates is available. Modern techniques such as molecular modeling and molecular dynamics methods, based on already identified 3D structures homologous to the studied protease enzymes, could be applied for the prediction of its substrate specificity and potent functional substrates in the proteome. The identification of novel proteases and the elucidation of the physiological functions of already identified plant proteases will certainly contribute to the development of modern science and biotechnology in general [18,139,140].

## Figures and Tables

**Table 1:** Proteases involved in ETI responses and PCD.

Plant Species	Plant Protease	Family	Subcellular Localization after Infection	Pathogen	Identified Substrates	Is Inhibited by Effector	Function/Phenotype	Ref.
*A. thaliana*	AtMC1, AtMC2	Cys, C14B	Cytoplasm, Nucleus	*Pst*DC3000	ND	-	Suppression of hypersensitive cell death response upon infection with avirulent pathogen, AtMC1 and AtMC2 antagonistically control *lsd1* runaway cell death	[32]
	AtMC9	Cys, C14B	Nucleus, Cytosol, Apoplast	*-*	GRI protein, PEPCK1, AtSerpin1	-	Effector of PCD activation, xylem cell death, degradation of vessel cell contents after vacuolar rupture	[70,71,72,73]
	CathB	Cys, C1A: CathB-like	Vacuole, Apoplast	*Pst*DC3000	ND	-	Required for the HR and disease resistance induced by non-host bacterial pathogens, positive regulatory role in senescence	[33]
	RD21	Cys, C1A: CathL-like	ER vesicles, Vacuole	*B. cinerea*	ND	-	‘Pro-death’ signal activated during elicitation of cell death, targeted by plant AtSerpin1, AtWSCP; processed by PttMC13 and PttMC14	[74,75,76]
	RD19A	Cys, C1A: CathL-like	Vacuole, Nucleus	*Ralstonia solanacearum*	ND	-	RRS1-R-mediated resistance, inhibited by effector PopP2	[77]
	VPEs	Cys, C13	Vesicles, Vacuole	*Pst*DC3000	Storage proteins (12S globulins and 2S albumins)	-	Activate vacuolar enzymes and disintegrate the vacuolar membrane to release hydrolytic enzymes during PCD, involved in the HR elicited by infection with TMV	[17,63,78]
	AtCEP1	Cys, C1A: CathL-like	ER	*Erysiphe cruciferarum*	ND	-	Restriction of powdery mildew controlling late stages of compatible interaction including late epidermal PCD	[79]
	PBA1	Thr, T1B	Cytosol, Nucleus	*Pst*DC3000	ND	-	Caspase-3-like (DEVDase) activity in the vacuolar and plasma membranes proteasome-regulating membrane fusion	[17]
	AtSBT5.2 (a and b)	Ser, S8	Endosomes	*Pst*DC3000	ND	-	Independent of protease activity attenuation of MYB30-mediated HR	[80]
*Picea abies* H. Karst.	mcII-Pa	Cys, C14	Cytoplasm, Nucleus	*-*	TSN	-	Induces autophagy, which triggers PCD mechanisms during the terminal differentiation of embryonic suspensor cells, and participates in further development of PCD	[81,82]
*Solanum lycopersicum* L*.*	P69B	Ser, S8	Apoplast	*Phytophtora infestans* *P. syringae*	ND	Kazal-like inhibitors EPI1 and EPI10	Local apoplast surveillance, substrate of Sl2-, Sl3-MMP, positive regulator of PCD	[83,84]
	P69C	Ser, S8	Apoplast	*P. syringae*	LRP protein	-	LRP protein processing	[56]
	Sl2-,Sl3-MMPs	Metallo, M10A	Apoplast	*B. cinerea* *Pst*DC3000	P69B	-	Extracellular cascade of epidermal cell death	[84]
	RCR3	Cys, C1A: CathL-like	Apoplast	*C. fulvum*, *P. infestans*, *Globodera rostochiensis*	ND	Avr2, EPICs, Gr-VAP1	Extracellular defense; co-receptor to Cf-2 for effector recognition in the case of *C. fulvum*	[67,85]
	PIP1	Cys, C1A: CathL-like	Plasma membrane, Apoplast	*C. fulvum*, *P. infestans*, *P. syringae*	ND	Avr2, EPICs	Broad-range extracellular defense	[85,86]
	CYP1	Cys, C1A: CathL-like	Apoplast	TYLCV	ND	V2	Involved in hypersensitive response reactions	[87]
	C14	Cys, C1A: CathL-like	Apoplast	*P. infestans*	ND	EPICs, AVRblb2	Defense-related secretion in haustoriated plant cells	[88]
	Sl-SBT3	Ser, S8	Apoplast	*P. infestans*	ND	-	Caspase-3-like DEVDase activity, HR-like PCD induction	[89]
*Populus tremula x tremuloides*	PttMC13, PttMC14	Cys, C14B	Cytoplasmic aggregates	-	RD21, TSN, PASPA3	-	Type II metacaspases, AtMC9 homologues, involvement of stress granules in the metacaspase-TSN pathway and xylem vessel and fiber cells PCD, processing of RD21, TSN, PASPA3—postmortem autolytic processes	[90]
*Solanum tuberosum* L.	StSBTc-3	Ser, S8	Apoplast	*P. infestans*	ND	-	Caspase-3-like DEVDase activity, HR-like PCD induction	[89]
*Avena sativa* L.	Saspase	Ser, S8	Apoplast	*Cochliobolus victoriae*	RuBisCO	-	RuBisCO proteolysis in victorin-induced PCD, IETDase and LEHDase activities	[91]
*Nicotiana tabacum* L.	Phytaspase	Ser, S8	Cytosol, Apoplast	TMV	VirD2 from *Agrobacterium tumefasciens*	-	Activated in tobacco mosaic virus (TMV)-induced HR, VirD2 cleavage preventing protein transport to nucleus, VEIDase, IETDase, LEHDase, and VDVADase	[92]

ND–not defined**.**

**Table 2:** Proteases involved in MTI/ETI downstream pathways, SAR and ISR.

Plant Species	Plant Protease	Family	SUBCELLULAR LOCALIZATION	Pathogen	Function/Phenotype	Ref.
*S. lycopersicum*	Sl3-MMP	Metallo, M10A	Plasma membrane	*Pst*DC3000, *B. cinerea*	Enhanced resistance to *B. cinerea* and upregulated expression of defense-related genes	[93]
	SBT3-Sl	Ser, S8	Tomato vasculature	*Manduca sexta* larvae	Herbivore defense, involved in systemin processing and JA-mediated resistance response	[94]
*Gossypium babardense* L.	GbSBT1	Ser, S8	Plasma membrane, cytoplasm	*Verticillium dahliae*	Associated with JA signaling	[95]
*V. quinquangularis*	AP13	Asp, A1	-	*Pst*DC3000, Powdery mildew, *B. cinerea*	Promotion of the SA dependent signal transduction pathway, but suppression of the JA signal transduction pathway, enhanced callose deposition	[59]
*A. thaliana*	SBT3.3	Ser, S8	Apoplast	*Pst*DC3000	H_2_O_2_-inducible positive regulator of innate immunity operating upstream of the SA pathway, MPK activation, concurrent chromatin remodeling at SA-responsive genes	[35]
	OTS1, OTS2	Cys, C48	Nucleus	*P. syringae*	OTS1 and -2 negatively regulate SA biosynthesis restricting biosynthesis gene ICS1 expression and propose that de novo synthesis and SA-promoted degradation of OTS1/2 antagonistically adjust the abundance of this negative regulator depending on the level of pathogen threat	[96]
	CDR1	Asp, A1	ER and apoplast	*P. syringae*	Induction of a SA-dependent resistance response; could generate endogenous extracellular peptides that act as mobile signals for SAR	[52]
	AED1	Asp, A1	Apoplast	*P. syringae*	Induced locally (EDS1-independent) and systemically (EDS1-dependent) during SAR signaling and locally by SA, homeostatic mechanism to limit SAR, tradeoff between defense and plant growth	[97]
	TOP1, TOP2	Metallo, M3	TOP1–chloroplasts, TOP2—cytosol	*-*	Non-competitive SA-binding, mediate SA-dependent signaling and are necessary for the immune response to avirulent pathogens	[98]
Maize Black Mexican Sweetcorn (BMS-33)	MIR1	Cys, C1A	Maize midwhorl	Caterpillars *Heliothis virescens*, corn leaf aphids	Pr oteolysis of caterpillar peritrophic matrix, ET-dependent, long-distance transport signal	[99]

## Abbreviations

AED1	apoplastic EDS1-dependent protein 1
AP13	aspartate protease 13
AtCEP1	cysteine endopeptidase 1 from *A. thaliana*
AtICS1/2	isochorismate synthase1/2 from *A. thaliana*
AtMC1/2/9	metacaspase 1/2/9 from *A. thaliana*
AtPAL1–4	phenylalanine lyase1 to 4 from *A. thaliana*
AZI1	azelaic acid induced 1
BAK1	brassinosteroid insensitive 1-associated receptor kinase 1
BIK1	*Botrytis*-induced kinase 1
CDPK	calcium-dependent protein kinase
CDR1	constitutive disease resistance 1
CERK1	chitin elicitor receptor kinase 1
CYP1	cysteine protease 1
DAMP	damage-associated molecular pattern
DIR1	defective in induced resistance 1
EDS1	enhanced disease susceptibility 1
EFR	EF-Tu receptor
ER	endoplasmic reticulum
ET	ethylene
ETI	effector-triggered immunity
FLS2	flagellin-sensing 2
HR	hypersensitive response
ISR	induced systemic resistance
JA	jasmonic acid
LRR	leucine-rich repeats
LSD1	lesion simulating disease 1
MAMPs	microbe-associated molecular pattern
MAPK	mitogen-activated protein kinase
mcII-Pa	metacaspase type II from *P. abies*
MeSA	methylsalicylic acid
MTI	MAMP-triggered immunity
NBS	nucleotide-binding site
NLR	NOD-like receptor
NLS	nuclear localization signal
NPR1/3/4	nonexpresser of PR genes 1/3/4
OTS1/2	overly tolerant to salt 1/2
OXI1	oxidative signal-inducible 1 kinase
PAMP	pathogen-associated molecular pattern
PCD	programmed cell death
PEPCK1	phosphoenolpyruvate carboxykinase 1
PIP1	*Phytophthora*-inhibited protease 1
PLCP	papain-like cysteine protease
PR	pathogenesis-related
PRR	pattern-recognition receptors
PstDC3000	*Pseudomonas syringae* pv. *tomato* DC30000
PTGS	post-transcriptional gene silencing
RALF23	rapid alkalinization factor 23
RD21	responsive to dehydration 21
RDR2	RNA-dependent RNA polymerase 2
RISC	RNA-induced silencing complex
RITS complex	RNA-induced transcriptional silencing complex
RLK	receptor-like kinase
RLP	receptor-like protein
RNA	ribonucleic acid
RNI	reactive nitrogen intermediate
ROS	reactive oxygen species
RRS1-R	Resistant to *R. solanacearum* 1-R
RuBisCO	ribulose-1,5-bisphosphate carboxylase/oxygenase
SA	salicylic acid
SAG12	senescence-associated gene 12
SAR	systemic acquired resistance
SBT	subtilase
Sl2/3-MMPs	matrix metalloprotease 2/3 from *S. lycopersicum*
SUMO1/2	small ubiquitin-related modifier 1/2
TGS	transcriptional gene silencing
TMV	tobacco mosaic virus
TOPs	thimet oligopeptidases
TSN	Tudor staphylococcal nuclease
VPE	vacuolar processing enzyme
WSCP	water-soluble chlorophyll protein
